# Perianesthetic mortality in English Bulldogs: a retrospective analysis in 2010 – 2017

**DOI:** 10.1186/s12917-022-03301-9

**Published:** 2022-05-25

**Authors:** Ayako Oda, Wen Hui Wang, Amanda K. Hampton, James B. Robertson, Lysa P. Posner

**Affiliations:** 1 Veterinary Anesthesiology Consultant, Tokyo, Japan; 2 Department of Molecular and Biomedical Sciences, College of Veterinary Medicine, North Carolina State University, Raleigh, NC USA; 3 College of Veterinary Medicine, North Carolina State University, Raleigh, NC USA

**Keywords:** English Bulldog, Brachycephalic, Peri-anesthetic mortality, Respiratory, Obstructive airway, Veterinary anesthesia

## Abstract

**Background:**

Many veterinarians consider English Bulldogs to have a greater perianesthetic mortality risk. The aims of this study were to 1) determine total and anesthesia-related, perianesthetic mortality (PAM) rates in English Bulldogs (EB), 2) identify potential risk factors associated with mortality in EB, and 3) determine the difference in the perianesthetic mortality rates between EB, other-brachycephalic breeds (OB), and non-brachycephalic breeds (NB).

Records from EB that were anesthetized between 2010 and 2017, were investigated. OB and NB were enrolled to match with each EB based on a procedure and age from the study period. Data collected in EB included: age, ASA status, weight, procedure types, anesthetic and analgesic management, anesthetic duration, anesthetic recovery location, and cause of death. Age and cause of death were determined from OB and NB. Fisher’s exact test was used to compare PAM rate and age in EB, OB, and NB. Mann–Whitney U test was used to compare EB survivor and EB non-survivor. Logistic regression models were used to identify factors and odds ratio (OR) associated with PAM in EB.

**Result:**

Two hundred twenty nine EB, 218 OB, and 229 NB were identified. The total and anesthesia-related PAM rates in EB were 6.6 and 3.9%, respectively. EB had a greater total PAM rate compared with OB (*p* = 0.007). ASA status was different between survivors and non-survivors in EB (*p* < 0.01). Risk factors identified regardless of the cause of death were premedication with full μ opioids (OR = 0.333, *p* = 0.114), continuous infusion of ketamine post-operatively (OR = 13.775, *p* = 0.013), and acepromazine administration post-operatively (OR = 7.274, *p* = 0.004). The most common cause of death in EB was postoperative respiratory dysfunction (87.5%).

**Conclusion:**

Total and anesthesia-related mortality in EB is considerable. Most deaths in EB occurred during the postoperative period secondary to respiratory complications.

**Supplementary Information:**

The online version contains supplementary material available at 10.1186/s12917-022-03301-9.

## Background

Many dogs from brachycephalic breeds have anatomical abnormalities such as stenotic nares, elongated soft palate, everted laryngeal saccules, and hypoplastic trachea, which together are referred to as brachycephalic obstructive airway syndrome (BOAS). Due to these abnormalities, brachycephalic dogs were widely considered to have a greater risk of perianesthetic mortality [1]. Recently, Gruenheid et al. [2] reported that brachycephalic dogs were more likely to experience perianesthetic complications compared with non-brachycephalic dogs, however brachycephaly is not a reported risk factor for perianesthetic mortality [3–12].

Confusingly, the umbrella term “brachycephalic breed”, is not standardized and each investigation includes different breeds such as; English Bulldog (EB), French Bulldog, Pug, Boston Terrier, Shih Tzu, Cavalier King Charles Spaniel, Chihuahua, and Boxer. In the authors’ experience, EB has a greater risk of perianesthetic complications including death compared with other brachycephalic breeds.

Thus, the aims of this study were 1) to determine perianesthetic mortality rate in EB, 2) to determine the difference in the perianesthetic mortality rates between EB, other brachycephalic breeds (OB), and non-brachycephalic breeds (NB), and 3) to identify risk factors associated with mortality in EB. It was hypothesized that EB have greater perianesthetic mortality rate compared with the rate in other brachycephalic breeds or non-brachycephalic breeds.

## Results

### Animals identified

Data were collected from 229 EB with patient matches for 218 OB and 229 NB. Breeds included in OB were French Bulldog (*n* = 44), Pug (*n* = 39), Shih Tzu (*n* = 35), Boxer (*n* = 35), Boston Terrier (*n* = 31), Cavalier King Charles Spaniel (*n* = 19), and Pekingese (*n* = 15). These breeds were included based on their brachycephalic head conformation [13]. Non-brachycephalic breeds are summarized in Table 1.

**Table 1:** List of non-brachycephalic breeds matched with each English Bulldog based on a procedure (primary priority) and age (secondary priority)

Breed	
Mixed-breed (*n* = 38)	Airedale Terrier (*n* = 1)
Labrador Retriever (*n* = 34)	Australian Heeler (*n* = 1)
American Staffordshire Terrier (*n* = 11)	Australian Shepherd (*n* = 1)
Golden Retriever (*n* = 10)	Basset Hound (*n* = 1)
Great Dane (*n* = 9)	Bedlington Terrier (*n* = 1)
Miniature and Toy Poodle (*n* = 8)	Belgian Malinois (*n* = 1)
Yorkshire Terrier (*n* = 8)	Belgian Tervuren (*n* = 1)
American Cocker Spaniel (*n* = 7)	Bernese Mountain Dog (*n* = 1)
Shar-Pei (*n* = 6)	Biewer Terrier (*n* = 1)
Chihuahua (*n* = 4)	Border Collie (*n* = 1)
German Shepherd (*n* = 4)	Bull Terrier (*n* = 1)
Rottweiler (*n* = 4)	Cane Corso (*n* = 1)
Beagle (*n* = 3)	Cavachon (*n* = 1)
Bichon Frise (*n* = 3)	Chesapeake Bay Retriever (*n* = 1)
Collie (*n* = 3)	Chinese Crested (*n* = 1)
Dachshund (*n* = 3)	Chow Chow (*n* = 1)
Doberman Pinscher (*n* = 3)	Clumber Spaniel (*n* = 1)
Irish Setter (*n* = 3)	Coton de Tulear (*n* = 1)
Jack Russell Terrier (*n* = 3)	Hound (*n* = 1)
Norwich Terrier (*n* = 3)	Greyhound (*n* = 1)
Australian Cattle Dog (*n* = 2)	Lakeland Terrier (*n* = 1)
English Setter (*n* = 2)	Manchester Terrier (*n* = 1)
Great Pyrenees (*n* = 2)	Mastiff (*n* = 1)
Italian Greyhound (*n* = 2)	Miniature Pinscher (*n* = 1)
Maltese (*n* = 2)	Old English Sheepdog (*n* = 1)
Miniature and Standard Schnauzer (*n* = 2)	Pointer (*n* = 1)
Papillon (*n* = 2)	Pembroke Welsh Corgi (*n* = 1)
Pomeranian (*n* = 2)	Samoyed (*n* = 1)
Portuguese Water Dog (*n* = 2)	Siberian Husky (*n* = 1)
Rhodesian Ridgeback (*n* = 2)	Spinone Italiano Dog (*n* = 1)
Saint Bernard (*n* = 2)	Standard Poodle (*n* = 1)
Silky Terrier (*n* = 2)	Tamaskan (*n* = 1)
Vizsla (*n* = 2)	Weimaraner (*n* = 1)
West Highland White Terrier (*n* = 2)	Woloitzcuintli (*n* = 1)

### Group demographics

The median (range) age of EB, OB, and NB was 3 (0.25 – 14), 5 (0.17 – 16), and 5 (0.2 – 13) years old, respectively. EB was significantly younger than OB (*p* = 0.0064) or NB (*p* = 0.0025).

### Perianesthetic mortality rates

The total mortality rate within EB (not accounting for multiple anesthetic episodes) was 6.6% with 95% confidence interval of (3.3%, 9.8%). The rate within episode (not accounting for patients receiving multiple anesthetic episodes) was 4.4% with a 95% confidence interval of 2.2%, 6.5%).

The perianesthetic mortality rates of EB, OB, and NB are shown in Table 2. Due to the lack of anesthesia-related deaths in NB, it is not possible to estimate the exact difference (odds ratio (OR) not available).

**Table 2:** Perianesthetic mortality rates in English Bulldog, other brachycephalic dogs, and non-brachycephalic dogs

	**EB (*n* = 229)**	**OB (*n* = 218)**	**NB (*n* = 229)**
Total deaths	15 (6.6%)	3 (1.4%)	4 (1.8%)
Odds ratio compared with EB	-	OR = 5.01 (*p* = 0.007)^*^	OR = 3.93 (*p* = 0.017)
Anesthesia-related deaths	9 (3.9%)	2 (0.92%)	0 (0%)
OR compared with EB	-	OR = 4.507 (*p* = 0.06241)	OR not available^a^

*EB* English Bulldog, *NB* Non-brachycephalic breeds, *OB* Other-brachycephalic breeds, *OR* Odds ratio

^*^Statistically significant at *p* value < 0.01

^a^OR not available due to lack of anesthesia-related death in NB, though the expected rate of anesthesia-related death to be less than 0.3%

### Survivors and non-survivors in English Bulldogs

Demographics of survivors and non-survivors in EB are shown in Table 3. Of the EB survivors, there were 43 intact males, 74 castrated males, 24 intact females, 71 spayed females, and one hermaphrodite. Of the EB non-survivors, there were 5 intact males, 4 castrated males, 2 intact females, and 4 spayed females.

**Table 3:** Demographics of survivors and non-survivors in English Bulldog

	**EB Survivors**		**EB Non-survivors**		***p*-value**
Age (years)	3 (0.25 – 14)	*n* = 214	4.1 ± 3.4	*n* = 15	0.948
Weight (kg)	24.3 (4.1 – 57.9)	*n* = 209	25.3 ± 7.0	*n* = 15	0.599
ASA physical status	2 (1 – 4)	*n* = 193	3 (2 – 4)	*n* = 12	< 0.01^*^
Anesthetic duration (hours)	3 (0.5 – 10)	*n* = 214	2.5 (0.5 – 4.5)	*n* = 15	0.263
Number of anesthetic episode	1 (1 – 23)	*n* = 214	1 (1 – 3)	*n* = 15	0.747

*ASA* American Society of Anesthesiologists, *EB* English Bulldog

^*^ASA physical status statistically different between survivors and non-survivors in EB (*p* < 0.05)

Risk factors evaluated are shown in Table 4. For Outcome 1 (survivors vs. non-survivors), the risk factors associated with a decreased OR of death included premedication with full µ opioids (PM full μ opioids) (OR = 0.333, *p* = 0.114). Risk factors associated with an increased OR of death included, ketamine continuous rate infusion (CRI) during the recovery period (RM Keta CRI) (OR = 13.775, *p* = 0.013), or acepromazine administration during the recovery period (RM Ace) (OR = 7.274, *p* = 0.004).

**Table 4:** Different potential risk factors evaluated between English Bulldog survivors and English Bulldogs which died from an anesthesia-related cause

	**EB Survivors**		**EB Non-survivors**			
Category of non-survivors	-		EB-AD		EB-ND	
Emergency admission	52	*n* = 214	6	*n* = 9	3	*n* = 6
Respiratory co-morbidity	88	*n* = 214	9	*n* = 9	1	*n* = 6
Cardiac co-morbidity	26	*n* = 214	2	*n* = 9	2	*n* = 6
GI co-morbidity	43	*n* = 214	5	*n* = 9	1	*n* = 6
Neurological co-morbidity	22	*n* = 214	0	*n* = 9	2	*n* = 6
Pregnancy status	10	*n* = 214	1	*n* = 9	0	*n* = 6
Anti-nausea drugs	78	*n* = 214	8	*n* = 9	1	*n* = 6
Prokinetic drugs	60	*n* = 214	7	*n* = 9	0	*n* = 6
Anti-acid drugs	74	*n* = 206	5	*n* = 9	0	*n* = 4
PM Full µ opioids	143	*n* = 204	3	*n* = 8	2	*n* = 4
PM Non-Full µ opioids	33	*n* = 204	0	*n* = 8	2	*n* = 4
PM Dexmedetomidine	65	*n* = 204	0	*n* = 8	0	*n* = 4
PM Midazolam	3	*n* = 204	1	*n* = 8	0	*n* = 4
PM Alfaxalone	2	*n* = 204	1	*n* = 8	0	*n* = 4
PM Acepromazine	6	*n* = 204	0	*n* = 8	0	*n* = 4
IND Propofol	197	*n* = 214	8	*n* = 9	4	*n* = 6
IND No Propofol	17	*n* = 214	1	*n* = 9	2	*n* = 6
MAINT Inhalant	197	*n* = 204	7	*n* = 8	4	*n* = 4
MAINT TIVA	5	*n* = 204	1	*n* = 8	0	*n* = 4
MAINT Both	2	*n* = 204	0	*n* = 8	0	*n* = 4
IA Full µ opioids	111	*n* = 205	7	*n* = 8	2	*n* = 4
IA Non-Full µ opioids	26	*n* = 205	2	*n* = 8	0	*n* = 4
IA Ketamine CRI	10	*n* = 204	1	*n* = 8	0	*n* = 4
IA Lidocaine CRI	17	*n* = 204	2	*n* = 8	0	*n* = 4
Local block	75	*n* = 204	4	*n* = 8	1	*n* = 4
Fluid bolus	160	*n* = 204	5	*n* = 8	2	*n* = 4
Anticholinergic	60	*n* = 204	2	*n* = 8	1	*n* = 4
Inotropic	46	*n* = 204	4	*n* = 8	1	*n* = 4
Vasopressor	5	*n* = 204	0	*n* = 8	0	*n* = 4
Suction	51	*n* = 204	1	*n* = 8	0	*n* = 4
RM Full µ opioids	76	*n* = 204	4	*n* = 7	0	*n* = 4
RM Non-full µ opioids	64	*n* = 204	4	*n* = 7	0	*n* = 4
RM Ketamine CRI	4	*n* = 204	2	*n* = 7	0	*n* = 5
RM Lidocaine CRI	1	*n* = 204	0	*n* = 7	0	*n* = 4
RM Acepromazine	33	*n* = 183	6	*n* = 7	1	*n* = 4
RM Dexmedetomidine	20	*n* = 204	0	*n* = 7	0	*n* = 4
RM NSAIDs	95	*n* = 211	1	*n* = 7	0	*n* = 4
RM Reversals	23	*n* = 204	0	*n* = 7	0	*n* = 4
Recovery place (IMC or ICU)	148	*n* = 214	8	*n* = 8	2	*n* = 2
Oxygen therapy	62	*n* = 214	6	*n* = 9	1	*n* = 4
Perianesthesia regurgitation	33	*n* = 214	4	*n* = 9	0	*n* = 6
Perianesthesia corticosteroid	63	*n* = 214	4	*n* = 9	1	*n* = 6

*EB* English Bulldog, *EB-AD* English Bulldogs which died from anesthesia-related cause, *EB-ND* English Bulldogs which died from non-anesthetic cause, *GI* Gastrointestinal, *PM* Premedication, *IND* Induction, *MAINT* Maintenance, *IA* Intra-anesthetic, *RM* Recovery medication, *IMC* Intermediate care unit, *ICU* Intensive care unit

For Outcome 2 (EB-AD, anesthesia-related deaths in EB vs. EB-ND, non-anesthetic deaths in EB), the potential risk factors identified were; whether the procedure was interventional in nature, acepromazine given in recovery (RM Ace), or gastrointestinal (GI) co-morbidity. The *p*-values for each of these in this model is 1 and the ORs are inestimable due to complete separation introduced by combinations of these factors.

Due to the small number of EB in each category for Outcome 3 (euthanized (*n* = 6) vs. cardiopulmonary arrest (CPA) (*n* = 9)) similar to Outcome 2, no statistical analysis was performed for Outcome 3.

### Number of anesthetic episodes and procedure types

Total number of anesthetic episodes for EB (*n* = 229) was 344 episodes. Of these, 53 EB (50 survivors and 3 non-survivors) underwent more than one episode of anesthesia during the study period. A total of 441 procedure types were performed on EB within the following categories: respiratory (*n* = 78), abdominal (*n* = 55), interventional (*n* = 140), bone (*n* = 45), and minor procedure (*n* = 123). Procedure types performed on EB non-survivors (at the time of death if there were multiple anesthetic episodes) were respiratory (*n* = 5), abdominal (*n* = 3), interventional (*n* = 5), minor (*n* = 5), and bone procedures (*n* = 0). The number of anesthetic episodes or procedure types was not a risk factor for perianesthetic mortality in EB.

### Deaths in English Bulldogs, other brachycephalic breeds, & non-brachycephalic breeds

Anesthesia related deaths in EB (*n* = 9) are summarized in Table 5. Six EB-AD were admitted to the hospital via the emergency service. EB-AD 2, 3, 5, 8, and 9 did not receive premedication prior to induction of anesthesia due to various clinical decisions including avoiding reduction of laryngeal motion caused by a drug during functional laryngeal exam. EB-AD 4 and 8 were anesthetized previously (during the same hospitalization) for laparotomy, and surgical correction of BOAS and temporary tracheostomy tube placement, respectively. Six of seven EB-AD were sedated with acepromazine during hospitalization. Among EB-ND (*n* = 6), 1 (16.7%) died intraoperatively due to fatal hemorrhage from surgical complication, and 5 (83.3%) were euthanized considering quality of life after diagnostic procedures were completed.

**Table 5:** Details on anesthesia-related death in English Bulldogs

EB-AD	Age (years)	ASA status	Anesthetic episodes	Procedures	Peri-anesthesia regurgitation	Peri-anesthesia O_2_ therapy	Post-anesthesia sedation	Time and cause of death
1	4.6	3	1	ophthalmologic	N	Y	Y (ace)	Post; CPA (respiratory)
2	1.3	3	1	BAS	Y (pre) (vomit, post)	Y	Y (ace)	Post; CPA (respiratory)
3	1	2	1	BAS, urogenital	Y (post)	Y	Y (ace)	Post; CPA (respiratory)
4	10.8	4	2	laparotomy	N	Y	Y (ace)	Post; CPA (respiratory)
5	3.7	3	1	Caesarean section	N	N	-	Intra; CPA (cardiac)
6	0.45	-	1	BAS	-	Y	-	Post; euthanasia (respiratory)
7	3.1	4	1	BVP, BAS	Y (post)	N	Y (ace)	Post; CPA (respiratory)
8	7.6	4	3	FLE	Y (pre)	Y	Y (ace)	Post; CPA (respiratory)
9	9.4	3	1	laparotomy	N	N	N	Post; CPA (unknown)

*EB-AD* English Bulldogs which died from anesthesia-related cause, *ASA* American Society of Anesthesiologists, *O*_*2*_ Oxygen, *N* No, *Y* Yes, *Pre* Preanesthetic, *Intra* Intra-anesthetic, *Post* Postanesthetic, *M* Male, *MC* Castrated male, *F* Female, *FS* Spayed female, *BAS* Brachycephalic airway surgery, *BVP* Balloon valvuloplasty, *FLE* Functional laryngeal exam, *IMC* Intermediate care unit, *ICU* Intensive care unit, *ace* acepromazine, *CPA* Cardiopulmonary arrest; -, data not available

Two anesthesia-related deaths were noted in OB (one post-anesthetic CPA and one euthanasia due to aspiration pneumonia). One OB was euthanized for non-anesthetic related reason intraoperatively. None of the deaths in NB were anesthesia related. All NB were euthanized due to poor prognosis of their diseases.

## Discussion

The total perianesthetic mortality rate in EB in this study was 6.6% (15/229) of which anesthesia-related mortality rate was 3.9% (9/229). Even though there was no statistical difference between the groups for anesthesia-related mortality rates, EB (3.9%) and OB (0.92%) or NB (0%), the authors consider these percentage differences to be clinically relevant. Similarly, the total rates for perianesthetic mortality between the groups (EB: 6.6%, OB:1.4%, NB:1.8%) identified only a statistical difference between EB and OB, the authors find the percentage differences compelling. It is likely that this study is underpowered.

Brachycephalic breeds have long been suspected to have increased anesthetic risks because of brachycephaly that predispose them to develop BOAS. BOAS can result in respiratory (laryngeal collapse) and GI complications (gastroesophageal regurgitation, GER) because of greater sub-atmospheric pressure during inspiration to overcome progressive and chronic upper airway obstruction [14]. A prospective study, which performed endoscopic examination of brachycephalic dogs with BOAS, revealed that 97.3% (71/73) had esophageal, gastric, and/or duodenal abnormalities [15]. Conscious, unmedicated, brachycephalic canine breeds including EB have lower arterial partial pressure of oxygen and greater arterial partial pressure of carbon dioxide than non-brachycephalic breeds based on arterial blood gas analysis at rest in room air [16]. Not surprisingly, many studies have shown that clinical signs and quality of life improve after surgical correction of BOAS; however, complete resolution of clinical signs may not occur [17–21]. It is postulated that preemptive correction of stenotic nares (and elongated soft palate if needed) in younger EB may delay development and progression of BOAS with understanding that the nasal resistance significantly contributes to total airway resistance [22]. All of these anatomic and physiologic differences likely contribute the differences in mortality rate identified in this study.

Three risk factors were detected for Outcome 1, whether the dog survived to discharge, or died. Premedication with a full μ opioid was associated with a decreased OR of death. It was surprising that administration of full μ opioid as a premedication was associated with reduced risk of death in EB because, full μ opioids decrease lower esophageal sphincter pressure [23], increase incidence of GER in dogs [24], and cause nausea and vomiting [25], all of which can lead to aspiration. It is possible that this association may be skewed since five of eight English Bulldogs that died intentionally did not receive any premedication to facilitate surgical procedure (e.g. laryngeal function exam and Caesarian section). Therefore, it is unknown whether an “opioid-restricted” protocol would decrease mortality.

Ketamine CRI in recovery was identified as a positive risk factor mortality with an OR of 13.8. The dosage range of ketamine CRI for the dogs in this study was (2 mcg kg^−1^ min^−1^—10 mcg kg^−1^ min^−1^). At these subanesthetic dosages, respiratory depression, GI effects, or central nervous system depression would not be expected. The addition of ketamine as a CRI in the recovery period may be instituted; as part of multimodal analgesic strategy, when opioid analgesia alone is considered insufficient, or when opioid administration is limited (e.g. by surgeon request). Thus, it is possible that a dog receiving ketamine might have very high analgesic needs which could result in robust dosing of drugs which could result in excessive sedation. Alternatively, if requested to limit opioid use, an anesthetist may choose to use a ketamine CRI to help with pain management. Unfortunately, the retrospective design of this project makes it impossible to know the reasons for ketamine CRI administration and thus this study cannot determine how addition of ketamine CRI was associated with an increased perianesthetic mortality rate in EB.

Administration of acepromazine during recovery was also associated with an increased mortality risk, with an OR of 7.3. Acepromazine is a dopamine receptor antagonist, used for sedation. It has a long elimination half-life (7.1 h) in dogs [26] and is commonly used in veterinary medicine for patients requiring long duration sedation. Acepromazine is often recommended to patients with respiratory distress [27]. Alternatively, acepromazine is administered with full μ opioids to produce neurolept-analgesia, which increases the analgesia produced by the opioid. As with the ketamine CRI, it is impossible to retrospectively evaluate the decision to administer this drug. While acepromazine does not cause significant respiratory depression or GI complications [26], it is possible that increased laryngeal muscle relaxation in dogs with GER, increased the risks of aspiration.

Outcome 2 evaluated the risk of death in EB from anesthesia related vs non-anesthesia related causes. While risk factors were identified (GI comorbidity, interventional procedures, and acepromazine given during recovery) the model was unable to provide OR. Statistically, this suggests that the sample size in each group was too small (underpowered) to estimate relative probability with confidence. In addition to implicated risk factors identified, all EB-AD in this study had pre-existing respiratory comorbidity (BOAS, exercise intolerance, respiratory distress, or history of aspiration pneumonia). To the authors’ knowledge, no study has evaluated this question to date.

In addition to the risk factors assessed in this study, there are other factors that might contribute to anesthetic risk that could not be evaluated via a retrospective study. It is likely that the severity of BOAS is a risk factor, but only 27% of dogs had a complete upper airway evaluation. Comparison of severity of BOAS with morbidity and mortality associated with anesthesia would likely be informative. Similarly, other anatomical abnormalities reported in EB, such as protrusion of nasopharyngeal turbinate [28], bronchial collapse [20], or the incidence of GER was unknown in most of the EB in this study.

Another risk factor, which was not evaluated in the current study, is pharyngeal myopathy. English Bulldogs have been studied as a natural model for obstructive sleep apnea in humans, and structural as well as functional changes in upper airway dilator muscles due to chronic obstructive respiration have been documented on histopathology [29], magnetic resonance imaging [30], and electromyography [31]. The sequela of the myopathy is decreased activity of upper airway dilator muscles during sleep, especially during rapid eye movement (REM) sleep, and this contributes to upper airway obstruction and hypoxemia. The effect of general anesthesia or common drugs used during a perianesthetic period on the activity of these muscles is unclear. Presence and severity of pharyngeal myopathy in other brachycephalic dogs have not been studied. Finally, it is possible that this is a complex subject with many factors involved, such that data from 229 EB may be too small to determine multiple risk factors for the greater perianesthetic mortality rate.

The result from the current study suggests that American Society of Anesthesiologists (ASA) physical status is not a good predictor for perianesthetic survival in EB. When EB survivors and non-survivors were compared, ASA physical status was found to be lower in survivors. Increasing ASA physical status has repeatedly been associated with mortality [3, 4, 7–10, 12] including one study specifically looking at brachycephalic breeds [2]. One possible reason that EB had higher mortality with lower ASA status is that many EB with moderate to severe BOAS have surgery to correct the airway defects early in life. These dogs are generally young and metabolically healthy, so are often assigned a low ASA score, even though the risks associated with their airways and GER likely place them at higher risk than is recorded. There is significant interobserver variability [32] when assigning ASA physical status.

Most EB that had anesthetic related deaths, 7/8 were caused by sequelae from respiratory complications within 48 h of postoperative period. While these findings were not statistically significant, the authors find the results clinically relevant. Gross regurgitation was observed in 4 of EB-AD, but it is possible that silent GER also occurred. There is an association between upper airway obstruction and GER [33], and a recent study reported that the prevalence of anesthetic GER detected with esophageal pH probe was 60% (12/20) in brachycephalic dogs [34]. The administration of prokinetic drugs in 7/9 of EB did not appear to prevent mortality, nor was it identified as a risk factor. It is also possible that an acute upper airway obstruction had led to GER and caused aspiration pneumonia during hospitalization.

Ages of EB and NB were different, even with the efforts to match the cases. Since the procedure was considered the first priority in matching cases (e.g. airway surgery), this likely explains the difference. Laryngeal tieback surgery is more commonly performed in older NB whereas surgical correction of BOAS is performed in younger EB.

Multiple limitations exist in this study. First, because this was a retrospective study, perianesthetic management was not controlled, and management decisions from different clinicians may have affected the data. Clinician based decisions include; recovery location (i.e. some clinicians keep EB in an oxygen cage during recovery regardless of clinical need), pro-kinetic drugs, full vs partial mu opioids, etc.). As with all retrospective clinical studies, some data was missing, and the validity of the study is reliant on the accurateness of the information in the record (e.g. cause of death, severity of disease, etc.). Second, over 20% (53/229) of EB had multiple anesthetic episodes during the study period, and data were combined to include one original dog for statistical analysis. Furthermore, some factors, such as procedures and co-morbidities, were categorized for the same reason. These data handling made the interpretation of results more difficult. Interestingly, however, the number of anesthetic episodes was not identified as a risk factor. Third, because multiple procedures were performed within one anesthetic episode for many EB, a respiratory procedure was chosen as the priority for matching with NB to compare the perianesthetic mortality rates. This could have caused bias because EB is already compromised regarding its upper respiratory system compared with NB. In addition, matched NB and OB may have had other procedures performed or had multiple anesthetic episodes during the study period. Fourth, because more EB had surgical correction of BAOS, other upper airways surgeries were chosen for the match. These procedures are likely not equivalent. Fifth, data were collected from only one veterinary teaching hospital in the United States. Clinical styles develop at institutions for anesthesia, surgery, recovery, intensive care unit (ICU) etc. It is unknown if these results reflect the risk of EB anesthesia elsewhere. Finally, although approximately 7-year-worth of data were collected, sample size may be too small to identify a risk factor (type II error).

In conclusion, the total and anesthesia-related perianesthetic mortality rates in English Bulldogs at a veterinary teaching hospital were 6.6% and 3.9%, respectively. However, anesthesia-related perianesthetic mortality rates between English Bulldogs and other brachycephalic dogs or non-brachycephalic dogs were not statistically different. Almost all English Bulldogs that suffered anesthetic related deaths had respiratory complications in the postoperative period. Only ASA physical status was found to be different between survivors and non-survivors in English Bulldogs. Full mu opioids were associated with less mortality in EB, whereas a constant rate infusion of ketamine or administration of acepromazine during a recovery period was associated with increased mortality in EB.

## Material and methods

### Animals

Medical records at North Carolina State University Veterinary Teaching Hospital from January 1, 2010 to September 30, 2017, were examined, using an integrated computer search program, to identify any dog labeled as purebred EB. All EB were included if they were anesthetized during the study period. For comparison, a non-brachycephalic group (NB) and an other-brachycephalic group (OB) were created by enrolling animals from the same study period and matched with each EB based on a procedure (the first priority) and age (the second priority). If EB underwent multiple procedures within one anesthetic episode, any airway procedure was considered as the first priority for the match. Because many cases for surgical correction of BOAS were performed in EB, other upper airway procedures, such as cricoarytenoid lateralization (tieback), cleft palate repair, tonsillectomy, and soft palate or laryngeal mass removal, were used as procedure matches with surgical correction of BOAS in NB and OB. If no respiratory procedure was involved, NB and OB were matched with EB based on the most invasive procedure performed. If the exact procedure could not be matched, a procedure with similar invasiveness was chosen. Both NB and OB may have had other procedures performed during the same anesthetic episode.

### Data collection

From each EB, the following data were collected by reviewing electronic and hand-written records: age, sex, weight, the ASA physical status, emergency status at admission, duration of anesthesia, number of anesthetic episodes within the period for data collection, procedures, co-morbidities, status of pregnancy, anesthetic drugs administered throughout an anesthetic episode and during recovery period, GI drugs administered, interventions for hypotension, recovery location after anesthesia, use of oxygen cage during hospitalization, notation of regurgitation at home or during hospitalization, use of suction in the upper GI tract, and use of corticosteroid. For statistical analysis, procedures were categorized into 5 categories (respiratory, abdominal, interventional, bone, and minor), and an additional file shows the categorization in more detail [see Additional file 1: Appendix 1]. Categorization was also performed on co-morbidities (respiratory, cardiovascular, GI, and neurologic), opioid type (full µ opioids vs. non-full µ opioids), GI drug use (anti-nausea, prokinetic, and antacid), interventions for hypotension (fluid therapy, anticholinergic, inotropic, and vasopressor), and recovery location. Recovery location after anesthesia was categorized based on the level of care unit; continuously monitored (ICU, intermediate care unit, and emergency service) and intermittently monitored (general wards).

Three mortality outcomes were evaluated. Outcome 1 assessed whether the EB patient was discharged from hospital (survivor) or died during hospitalization (non-survivors) regardless of the cause of death. Outcome 2 assessed whether the EB patient died from a cause related to anesthesia (EB-AD) or died from a cause unrelated to anesthesia (EB-ND). Anesthesia-related death was considered as a death within 48 h after an anesthetic event unless a specific reason of the death unrelated to anesthesia was noted. Outcome 3 assessed whether the EB patient was euthanized or experienced CPA. If a dog had more than one episode of anesthesia, data were combined and reported as one dog. An average was determined for each numerical variable such as age and weight. If different anesthetic protocols were used or different procedures were performed on the same EB, all interventions and procedures were included even though they were not performed during the same anesthetic episode.

For each dog in the NB and OB groups, age and Outcome 1 and 2 were identified.

### Statistical analysis

Statistical analysis was performed using two different commercially available statistical software programs (GraphPad Prism 8, GraphPad Software Inc., CA, USA; R version 4.0.2, R Foundation for Statistical Computing, Vienna, Austria). The *p*-value was set as 0.05 or less unless specified. Numerical data were tested for normality with D'Agostino-Pearson normality test, and parametric data were reported with mean ± standard deviation (SD) whereas nonparametric data were reported with median (range). Mann–Whitney U test was used to compare characteristics of survivors and non-survivors in EB.

To estimate the perianesthetic mortality rate, a normal confidence interval was constructed around the point estimate. To examine potential risk factors, the selected variables were examined one-by-one with logistic regression then all variables with a *p*-value less than 0.10 were included in a full model for survival and a *p*-value less than 0.20 was considered sufficient when looking at anesthesia-related death. The full model was reduced by use of the Bayes’ Information Criteria. To compare mortality rates between EB, NB, and OB, Fisher’s exact test was used, and *p*-value was set as 0.01 to correct for multiple testing and keep the α rate at 0.05.

## Supplementary Information

**Additional file 1: Appendix 1.** Procedure categorization.

## Data Availability

The datasets used and/or analyzed during the current study are available from the corresponding author on reasonable request.

## Abbreviations

BOAS: Brachycephalic obstructive airway syndrome
EB: English Bulldogs
NB: Non-brachycephalic comparison group
OB: Other-brachycephalic comparison group
ASA: American Society of Anesthesiologists
GI: Gastrointestinal
EB-AD: English Bulldogs died from anesthesia-related causes
EB-ND: English Bulldogs died from non-anesthetic causes
CPA: Cardiopulmonary arrest
SD: Standard deviation
OR: Odds ratio
CRI: Constant rate infusion
GER: Gastroesophageal reflux
REM: Rapid eye movement
