# Supplementary material for: Perianesthetic mortality in English Bulldogs: a retrospective analysis in 2010 – 2017
Source: BMC Vet Res. 2022 May 25;18:198. doi: 10.1186/s12917-022-03301-9 (PMC9131676; doi:10.1186/s12917-022-03301-9)
Supplement: Supplementary file 1 — Additional file 1: Appendix 1. Procedure categorization. [file 12917_2022_3301_MOESM1_ESM.docx]

**Appendix 1: Procedure categorization**

| **Respiratory** | **Abdominal** | **Interventional** | **Bone/Head** | **Minor** |
| --- | --- | --- | --- | --- |
| Brachycephalic airway surgery  Tracheoscopy  Rhinoscopy  Bronchoalveolar lavage  Temporary tracheostomy placement  Endotracheal wash | Laparotomy | Transvenous cardiac procedure  Thoracoscopic procedure  Intrathoracic procedure  Diagnostic imaging  Endoscopic procedure  Radiation therapy | Orthopedic procedure  Hemilaminectomy  Tail amputation  Dental procedures  Total ear canal ablation  Maxillectomy  Mandibulectomy  Pinnectomy | Ophthalmologic procedures  Wound management  Biopsy/fine needle aspirate  Urogenital procedure  (no spay)  Cerebral spinal fluid tap  Esophageal feeding tube placement  Dermatologic procedure |
